# Medicinal plants in the cultural landscape of a Mapuche-Tehuelche community in arid Argentine Patagonia: an eco-sensorial approach

**DOI:** 10.1186/1746-4269-10-61

**Published:** 2014-08-26

**Authors:** Soledad Molares, Ana Ladio

**Affiliations:** 1 CONICET-Facultad de Ciencias Naturales, Universidad Nacional de la Patagonia San Juan Bosco, Ruta Nacional N° 259 km 16,41, Esquel 9200, Chubut, Argentina; 2 INIBIOMA, CONICET- Universidad Nacional del Comahue, Quintral 1250, Bariloche 8400, Río Negro, Argentina

**Keywords:** Patagonia, Argentina, Medicinal traditional knowledge, Organoleptic traits

## Abstract

**Background:**

The taste and smell of medicinal plants and their relation to the cultural landscape of a Mapuche-Tehuelche community in the Patagonian steppe was investigated. We assume that the landscapes as a source of therapeutic resources is perceived, classified and named according to different symbolic, ecological and utilitarian criteria which are influenced by chemosensorial appearance of medicinal plants which are valued by inhabitants.

**Methods:**

Information relating to the cultural landscape experienced by 18 inhabitants, all representing 85% of the families, in terms of medicinal plants, knowledge of species and their organoleptic perception was obtained through participant observation, interviews and free listing. The data were examined using cualitative and quantitative approach, including discourse analysis and non-parametric statistics.

**Results:**

Informants use 121 medicinal species, obtained from both wild and non-wild environments, most of which (66%) present aroma and/or taste. It was found that the plants with highest use consensus used for digestive, respiratory, cardio-vascular, analgesic-anti-inflammatory, obstetric-gynaecological and genito-unrinary complaints, have the highest frequencies of cites reporting flavor; and those with the highest frequencies relating to digestive, analgesic-anti-inflammatory and cultural syndromes present the highest frequencies of aroma. Flavor and/or aroma are interpreted as strong or soft, and the strongest are associated with treatment of supernatural ailments. Also, taste is a distinctive trait for the most of the species collected in all natural units of the landscape, while aroma is more closely associated with species growing at higher altitudes. The local pharmacopeia is also enriched with plants that come from more distant phytogeographical environments, such as the Andean forest and the Patagonian Monte, which are obtained through barter with neighboring populations. Herbal products are also obtained in regional shop. The practices of barter and purchase extend the limits of the landscape as a provider of therapeutic resources, improving the dynamics of its functions and structure, leading to more effective solutions to the various health needs that arise in the community.

**Conclusions:**

Herbal landscape perceived by the community exhibits notable eco sensorial and spatial heterogeneity. Local inhabitants’ sensorial interpretations play a role as heuristic tools in the recreation and redefinition of old and new available resources.

## Background

Cultural landscapes are intimately linked ecological-cultural systems, and therefore seen as biocultural phenomena [1]. They are the result of physical, biological, cultural and social diversity, and their structure is delineated by diverse learning and adaptation processes which are put into practice to deal with environmental circumstances [2]. Domestication processes, successional management, efficient use of environmental heterogeneity and other forms of interaction with plant components are examples of how humans shape their environment [3,4]. Cultural landscapes are also the result of particular cosmovisions, with symbolic meanings and values which transcend mere usefulness; they signify vital opportunities for nourishment, recreation and healing, among many other functions [5].

With respect to cultural landscapes as a source of therapeutic resources, Sõukand and Kalle [6] propose the concept of ‘herbal landscape’, referring to the area where the psycho-physical and emotional needs of individuals or social groups are generated and where treatments and prophylactic or healing measures can be taken. According to these authors, limits in terms of space are determined and demarcated as much according to geo-climatic factors as to socio-cultural ones. These limits are not absolute, but flexible, and allow the entry and exit of native and exotic plants, people, knowledge and processes, and are therefore functionally dynamic over time [7].

According to different authors [6,8], perception of the landscape as a domestic, therapeutic and agro-breeding area is found to be strongly influenced by the sensorial qualities of its components, and is established by the particular points of view of the human group which passes through, uses and reproduces it. However, this phenomenon has been little taken into account up to now as a focus of study [8]. It has been proposed that the sensorial appearance of plants, that is, the set of its characteristics of flavor, aroma, color, texture, shape etc., could act as a link between socially developed ideas and the plant world, influencing the people’s behavior in terms of group strategies for exploring habitats and the use of resources [9]. This is why sensorial stimuli play an important role in diet selection, medicine, religion, memory, sexuality, the relationship between human groups and their natural environment [9], especially in groups which are closely associated with their natural surroundings [10].

Previous work on this subject in Subantarctic Patagonia indicate that species with highest consensus of use have the most agreement in terms of their characteristic of taste and smell [8]. A specific study of gastrointestinal Patagonian forest plants found that stomach complaints are treated with delicate and sweet plants, that hepatic complaints are generally treated with bitter ones, and that plants used as laxatives are generally spicy [11]. Moreover, we found that the intensity of aroma and/or flavor perceived by people can influence the dosage and method of administration of the herbal preparation [8,11].

On the other hand, studies on environmental perception and the successional management of sub Antarctic forest by Mapuche communities in order to obtain medicinal plants have found that the emic landscape categories seem to be founded on the order given to the universe, relationships between the people and past phenomena, and on current, everyday life [11,12]. In addition, the diverse collective interpretations of sensorial signs have been related to the therapeutic and symbolic usefulness of the environmental units and their plants [8].

Knowledge of these aspects in arid zones is still very scarce, particularly in Patagonia. However, it is known that the wild aromatic and sapid plants of arid environments, and the cultural perceptions associated with them, play an important role within traditional medical systems [13-15], possibly, to some extent, in response to their high abundance and visibility in these ecosystems [16].

The rural populations living in arid Patagonia, which are mainly of Mapuche-Tehuelche origin, have developed numerous strategies for the use of natural resources which are often scarce and/or inaccessible due to the inhospitable context [17,18]. Among these strategies, the exploration and differential use of the natural surroundings, the ecological transformation of the land through, for example, cultivation, and the protection given to certain plants, are behaviors that demonstrate the strong cultural roots underlying the search and promotion of medicinal species [19,20]. For example, the exotic plants with a strong aroma and flavor that are cultivated in homegardens are valuable elements in the pharmacopeias of this region [11]. In relation to this, differential relevance of the domestic environments as providers of medicinal plants has been suggested, such as the peridomestic and the secondary ecosystems [21].

In this work with the Nahuelpan Mapuche-Tehuelche community of the Argentine Patagonian steppe, the cultural landscape as a source of medicinal plants (herbal landscape) are analyzed from an eco-sensorial approach. The sensorial characteristics of these plants and their relation to the perception, classification and use of the environment are investigated.

Our hypotheses were: 1) The cultural landscape associated with medicinal use of its plant resources (herbal landscape) is perceived, classified and named according to different symbolic, ecological and utilitarian criteria which are influenced by its sensorial appearance. 2) The cultural importance of the different units of the herbal landscape has an organoleptic pattern; it depends on the sensorial characteristics, relative importance and symbolic value of the medicinal species they are composed of. 3) The plants used for diverse therapeutic purposes have distinctive eco-sensorial features which are perceived and valued by inhabitants. 4) Species with high consensus of use and relative importance, in any environment and use category, have the highest consensus about its organoleptic characteristics (taste and/or aroma).

## Materials and methods

### Study area

**Figure 1:**
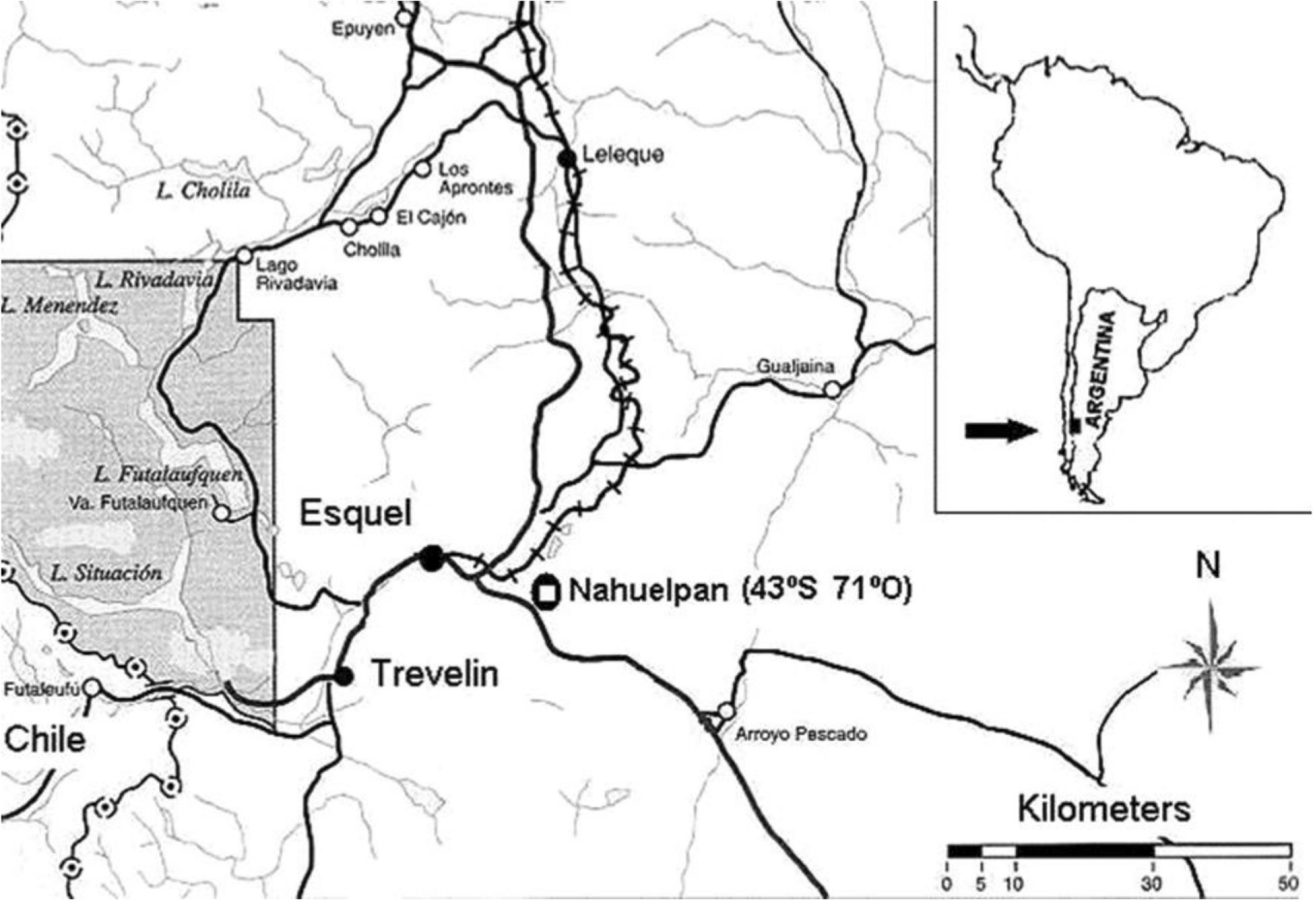
Map of the Mapuche-Tehuelche Nahuelpan community, Chubut Province, Argentina.

Nahuelpan is situated in the north west of Chubut province, Patagonia, Argentina (43° S, 71° W) (Figure 1). The general climatic characteristics are strong winds and frosts all year round, snow in winter and a dry season in summer. The average annual temperature is 8°C, with precipitation of approximately 400mm annually. Phytogeographically, this area is included in the occidental Patagonian district, Patagonia province [22]. The dominant vegetation is typical grass-bush steppe, with an abundance of graminaceous *Poa* spp., *Pappostipa* spp. and *Festuca* spp., subshrubs of *Mulinum spinosum* Cav. (Pers.), bushes of *Nassauvia glomerulosa* (Lag. ex Lindl.) D. Don*, N. axillaris* (Lag. ex Lindl.) D. Don*, Berberis microphylla* G. Forst., *Adesmia volckmannii* Phil.*, Nardophyllum bryoides* (Lam.) Cabrera, *Azorella monantha* Clos, *Senecio filaginoides* DC., and in some sectors *Corynabutilon bicolor* (Phil. ex K. Schum.) and *Schinus roigii* Ruiz Leal & Cabrera. Among the herbaceous plants the most notable are *Cerastium arvense* L., *Oenothera odorata Jack., Arjona tuberosa* Cav., *Euphorbia collina* Phil., *Plantago lanceolada* L., *Acaena pinnatifida* Ruiz & Pav., *Calceolaria* sp., *Rumex acetosella* L., among other plants. In areas close to “mallines” (wetlands of glacial origin) the woody plants *Discaria* spp., *Schinus patagonicus* (Phil.) I.M. Johnst. ex Cabrera and *Baccharis obovata* Hook. & Arn. grow, as well as the herbaceous *Mutisia retrorsa* Cav. and *Taraxacum officinale* G. Weber ex F.H. Wigg., among others. In the highest areas with southern exposure, or in the wet gullies, stunted *Nothofagus pumilio* (Poepp. & Endl.) Krasser forests grow [22].

### The Nahuelpan community

The Nahuelpan community is composed of 60 people, living in 21 homes. Land ownership is communal, under the category ‘indigenous reserve’, with the exception of two families who have a title deed to their properties. The area of indigenous reserve is 7500 hectares [23]. This population comes under the jurisdiction of the municipal government of Esquel (43900 inhabitants, town center 15km distant), department Futaleufú. Most inhabitants are Mapuches, and some are Tehuelches. The Mapuche native language is Mapuzungun (‘language of the land’), of the Nahuelpan linguistic tribe, but at present only 6% of the population are active speakers, and they are elderly and bilingual [23]. There are no speakers of the native Tehuelche language.

The principal economic activity is livestock raising, on a large scale, with sheep and goats bred for wool and meat production. The products originating from this activity satisfy only basic subsistence requirements.

One of the stations of the old narrow gauge railway “La Trochita” is found in this locality, and is of recognized value for national and international tourism, thus favoring the sale of knitted garments and regional foods (*tortas fritas,* jams and home made bread) to the tourists who arrive regularly for a visit.

The main public institution is the primary school, where cultural events take place such as workshops teaching the Mapuche language, veterinary medicine, vegetable gardening, spinning, wool dyeing and knitting, generally organized by extension organizations such as the National Institute of Farming Technology (INTA: *Instituto Nacional de Tecnología Agropecuaria*) or the Social Farming Program (PSA: *Programa Social Agropecuario*). Funeral wakes are also held in the school buildings, which function as a community center.

Most of the population dwells close to the school and train station, where the craft shop and a small museum dedicated to the history and indigenous culture of Patagonia are also situated. The remaining dwellings are located between one and fifteen kilometers from this center.

Inhabitants usually move around between houses, or go to the school or Esquel on horseback, on foot or by hitching a lift, since few own a vehicle. There is no public transport in the area. Nevertheless, very frequent visits are made to Esquel since most families have relatives living in this city.

The local medical system can be identified as Creole [24], since therapeutic criteria pertaining to the Mapuche culture [25], coexist with European elements, Christian symbolism, magic practices and the official medicine. The community has no health clinic, but a health worker who resides in the community visits the homes periodically and constitutes a link with the regional hospital in Esquel.

The absence of alternative work, the scarcity of land, which limits the size of the herds, and the lack of water, which restricts the development of agriculture, among other socio economic factors, converge and lead to an accelerated process of migration of the youngest community members, who move mainly to the cities of Esquel, Trelew (607 km) and Comodoro Rivadavia (595 km). Consequently, there are an elevated proportion of adults and elderly in the population.

### Collection of ethnobotanical data

Fieldwork was carried out within the framework of the principles established by the United Nations 1992 Rio de Janeiro Convention on Biological Diversity for the regulation of access to genetic resources and associated knowledge, protection of traditional knowledge and recognition of rights to intellectual property [26]. Authorization for this study was requested from local officials (tribal chief, health worker and headmistress of local school). In addition, informed consent was requested from each interviewee.

Information relating to the cultural landscape experienced by each informant in terms of medicinal plants, knowledge of species and their organoleptic perception was obtained through participant observation, free listing and semi-structured and in-depth interviews, considering that the concept of perception is equivalent to representation, as the externalization of what an individual perceives through all physiological pathways (biological, psychological and cultural senses) [27].

Sampling method consisted of a census of all households in the community. A family of each house was invited to participate in the study. Eighteen families agreed to participate, and one member of each family offered to be interviewed voluntarily. In total, 14 permanent inhabitants and 4 semi-permanent (individuals who live part of the week in Esquel) were interviewed, all representing 85% of the families.

We use a free listed technique for studied the richness of known and used medicinal species, and its Creole and Mapuzungun names. Through interviews we inquire the methods of preparation and administration of each species, procurement strategies (collection, cultivation, purchase and/or barter), and gathering environments. In the depth analysis of the discourses we approach to the importance of the general chemo-sensorial (aroma and taste) perception involved in the recognition, selection and use of medicinal plants, as well as other items of general interest.

In addition, walks with and without informants were carried out, during which reference material was collected which was placed in the BCR herbarium of the *Universidad Nacional del Comahue*. The botanical nomenclature used follows Zuloaga *et al*. [28]. The Mapuzungun nomenclature used follows Díaz Fernández [23].

### Data analysis

Analysis was quali-quantitative, following different methods used in the study of environmental perception to assess worldviews, sensations, values and opinions [27].

The nomenclature and biogeographical origin (native or exotic to Patagonia) of the medicinal species was checked by consulting Zuloaga *et al*. [28]. Total species richness and richness of botanical families was estimated using the total sum of species and families respectively. Use consensus for each species was calculated with the formula: number of informants who cited each species/total number of informants (N = 18) x 100 [8].

Using qualitative discourse analysis [27], opinions given by the informants regarding the intensity, characteristics and quality of the plant organoleptic sensations recognized were analyzed, and in particular, the significance of the plants and of their sensorial attributes as expressed in Mapuzungun. Following this, the perceptions of aroma and taste were classified according to the informants’ (emic) criteria.

The consensus on organoleptic characteristics (OC) was also estimated using their relative frequencies (total number of mentions of the organoleptic characteristic under consideration for the species *i* x 100/total number of informants who cite the species *i*) [29].

Following this, the medicinal uses were grouped in etic categories in order to enable quantitative comparisons to be made [25]: gastro-intestinal (GI), respiratory (RE), analgesic-anti-inflammatory (AAI), dermatological-cosmetic (DC), cultural syndromes (CUS), fever (F), cardio-vascular (CV), obstetric-gynaecological (OG), genito-urinary (GU), and other uses (endocrine, ophthalmic, etc.) (O). The numbers registered were compared between categories with the Chi squared test (*p* <0.05).

The analysis of the relation between the frequency of cites for each therapeutic use category and the frequency of organoleptic aroma cites was analyzed with the Spearman test (*p* <0.05).

The plant landscape units which provide medicinal plants were characterized according to informants’ precepts [27]. Aromatic and sapid species richness was compared with the richness of non aromatic and non sapid species for each unit, using the binomial test (*p* <0.05).

In order to compare the cultural importance of the different plant landscape units, the relative importance (RI) of each species was calculated per unit [30], using the following formula: RI = NBS + NP, where NBS is the number of body systems treated by a particular species (NBSS) divided by the number of body systems treated by the most versatile species (NBSVS); NP is the relation between the number of medicinal properties attributed to a given species (NPS) divided by the total number of properties attributed to the most versatile species (NPVS). Following this, in order to obtain weighted frequencies, the RI values were multiplied by the consensus of each species. The maximum value of this index is 200. The values thus obtained were compared between different landscape units with the Kruskal-Wallis test [8].

## Results and discussion

### Herbal landscape and eco-sensoriality: collection sites and their relation to the organoleptic characteristics of medicinal species

The Nahuelpan herb landscape is heterogeneous and includes areas which have been transformed to some extent, presenting different levels and types of relationship between inhabitants and the physical and natural components. In this landscape most medicinal plants are acquired by collection (78% of cites), and to a lesser extent by cultivation and harvesting (9%), and purchase or barter (13%) (*p* <0.05) (Table 1).

**Table 1:** Medicinal species used in the Mapuche-Tehuelche Nahuelpan community

**Species**	**Family**	**Common names**	**Origin**	**Unit of the landscape**	**Medicinal uses**	**Consensus**	**RI**	**RI**×**Consensus**	**Sensorial property**
*Gunnera tinctoria* (Molina) Mirb.	Gunneraceae	Nalca	Na	Water bodies/Purchase/barter	GI, RE, CV, AAI, CUS, OG, GU	83.3	1.9	156.3	TA
*Valeriana carnosa* Sm.	Valerianaceae	Ñamkulawen^b^	Na	Stony slopes	GI, RE, CV, AAI, DC, CUS, O, GU	66.7	1.9	127.3	TA, SM
*Artemisia absinthium* L.	Asteraceae	Ajenco, ajenco plateado, ajenco blanco	Ex	Anthropized areas	GI, RE, AAI, DC, CUS, F	72.2	1.7	119.8	TA, SM
*Adesmia boronioides* Hook. f.	Fabaceae	Paramela	Na	Stony slopes	GI, RE, AAI, DC, CUS, O, GU	66.7	1.7	112.9	TA, SM
*Fabiana imbricata* Ruiz & Pav.	Solanaceae	Palo piche	Na	Water bodies	GI, RE, CV, AAI, DC, CUS, OG, GU	72.2	1.5	111.6	TA, SM
*Neuropogon* sp.^a^	Parmeliaceae	Barba de piedra	Na	Stony slopes	GI, RE, CV, CUS, OG, GU	61.1	1.5	90.3	TA
*Equisetum bogotense* Kunth	Equisetaceae	Limpia plata	Na	Water bodies	GI, RE, CV, AAI, DC, CUS, F, GU	44.4	1.8	80.8	NO
*Erodium cicutarium* (L.) L’Hér. ex Aiton	Geraniaceae	Alfilerillo	Ex	Anthropized areas	GI, RE, CV, DC, CUS, F	50	1.6	78.4	NO
*Buddleja globosa* Hope	Buddlejaceae	Pañil^b^	Na	Purchase/barter	GI, AAI, DC, CUS, OG, O	38.9	1.8	68.1	TA, SM
*Acantholippia seriphioides* (A. Gray) Moldenke	Verbenaceae	Tomillo del campo	Na	Dry plates	GI, RE, CV, CUS, GU	44.4	1.3	56.1	TA, SM
*Marrubium vulgare* L.	Lamiaceae	Malva rubia	Ex	Anthropized areas	RE, F, O	61.1	0.8	50.7	TA
*Matricaria recutita* L.	Asteraceae	Manzanilla	Ex	Purchase	GI, AAI, CUS, O	38.9	1.2	47.7	TA, SM
*Baccharis sagittalis* (Less.) DC.	Asteraceae	Carqueja	Na	Water bodies	GI, AAI	83.3	0.6	51.1	TA, SM
*Grindelia chiloensis* (Cornel.) Cabrera	Asteraceae	Botón de oro	Na	Dry plates	RE, AAI, DC, CUS, F, O	38.9	1.1	43.3	SM
*Anarthrophyllum* sp.	Fabaceae	Nenew^b^ macho	Na	Dry plates	RE, OG, O	50	0.7	36.9	TA, SM
*Apium prostratum* Labill.	Apiaceae	Apio del campo	Na	Stony slopes	RE, AAI, F, O	44.4	0.9	38.4	TA, SM
*Ribes cucullatum* Hook. & Arn.	Grossulariaceae	Zarzaparrilla	Na	Water bodies	CV, DC, CUS, O	44.4	0.9	38.4	SA, AR
*Acaena splendens* Hook. & Arn.	Rosaceae	Cesped caballo	Na	Dry plates	RE, AAI, OG, GU	33.3	0.9	28.8	TA
*Oxalis* sp.	Oxalidaceae	Culle verde	Na	Stony slopes	RE, OG, F	38.9	0.8	32.3	TA
*Ruta chalepensis L.*	Rutaceae	Ruda	Ex	Cultivated areas	GI, AAI, CUS	27.8	0.9	25.6	TA, SM
*Tanacetum vulgare* L.	Asteraceae	Ajenco amarillo, ajenco, ajenco verde	Ex	Cultivated areas	GI, RE, AAI, F	27.8	1.0	29.0	TA, SM
*Sedum telephium* L.	Crassulaceae	Bálsamo	Ex	Cultivated areas	AAI, DC	33.3	0.6	20.5	NO
*Solanum crispum* Ruiz & Pav.	Solanaceae	Natre	Na	Purchase/barter	RE, F	44.4	0.5	23.2	TA
*Clinopodium darwinii* (Benth.) Kuntze	Lamiaceae	Té pampa, té de campo	Na	Stony slopes	GI, GU	55.6	0.4	24.0	TA, SM
*Urtica urens* L.	Urticaceae	Ortiga	Ex	Anthropized areas	CV, AAI, DC, CUS, OG	16.7	1.2	19.5	TA, SM
*Baccharis obovata* Hook. & Arn.	Asteraceae	Wawtru^b^	Na	Water bodies	AAI, DC	38.9	0.5	20.3	TA, SM
*Allium cepa* L.	Alliaceae	Cebolla	Ex	Purchase	RE, CUS, F	16.7	0.8	13.8	TA, SM
*Oxalis adenophylla* Gillies ex Hook. & Arn.	Oxalidaceae	Culle colorado	Na	Stony slopes	RE, DC, OG, F	22.2	0.9	19.2	TA
*Stillingia patagonica* (Speg.) Pax & K. Hoffm.	Euphorbiaceae	Trintri lawen^b^, mata perro	Na	Dry plates	GI, AAI, DC	16.7	0.8	13.8	TA
*Armeria maritima* (Mill.) Willd.	Plumbaginaceae	Piuke lawen^b^	Na	Dry plates	GI, CV, AAI	22.2	0.7	16.4	TA, SM
*Dysphania ambrosioides* (L.) Mosyakin & Clemants	Chenopodiaceae	Paico	Na	Anthropized areas	GI	38.9	0.4	15.5	TA, SM
*Mentha pulegium* L.	Lamiaceae	Poleo	Ex	Water bodies	GI	38.9	0.3	11.9	TA, SM
*Chuquiraga avellanedae* Lorentz	Asteraceae	Trayaw^b^, pincha bola, quilembay	Na	Dry plates	RE	33.3	0.5	16.3	TA
*Gossyphiantus lanuginosus* (Poir.) Hook. f.?	Amaranthaceae	Yerba del pollo	Ex	Purchase	GI	22.2	0.5	10.9	TA, SM
*Limonium brasiliense* (Boiss.) Kuntze	Plumbaginaceae	Guaycurú	Na	Dry plates	GI, RE, AAI, CUS	16.7	0.9	14.4	TA
*Maihuenia patagonica* (Phil.) Britton & Rose	Cactaceae	Chupasangre, tuna	Na	Dry plates	CV, AAI, DC	16.7	0.6	10.8	NO
*Melissa officinalis* L.	Lamiaceae	Torongil, melisa	Ex	Cultivated areas	GI, CV, CUS	27.8	0.6	18.0	TA, SM
*Mulinum spinosum* (Cav.) Pers.	Apiaceae	Nenew^b^	Na	Dry plates	RE, AAI, O	16.7	0.6	10.8	TA, SM
*Sambucus nigra* L.	Adoxaceae	Sauco	Ex	Cultivated areas	RE, AAI, F	27.8	0.6	18.0	TA
*Dysphania multifida* L.	Chenopodiaceae	Paico	Ex	Anthropized areas	GI, RE	22.2	0.5	11.6	TA, SM
*Plantago lanceolata* L.	Plantaginaceae	Siete venas, siete velos	Ex	Anthropized areas	AAI, DC	22.2	0.5	11.6	NO
*Centaurium cachanlahuen* (Molina) B.L. Rob.	Gentianaceae	Kachanlawen^b^, cachanlagua	Na	Stony slopes	RE, F	22.2	0.4	9.6	TA
*Corynabutilon bicolor* (Phil. ex K. Schum.) Kearney	Malvaceae	Monte moro	Na	Dry plates	RE, CUS, F	16.7	0.7	12.3	NO
*Eucalyptus* sp.	Myrtaceae	Eucalito	Ex	Purchase/barter	RE, CUS	16.7	0.5	8.7	TA, SM
*Linum usitatissimum* L.	Linaceae	Linaza, lino	Ex	Purchase	OG, F	16.7	0.5	8.7	TA
*Rosmarinus officinalis* L.	Lamiaceae	Romero	Ex	Purchase	GI, AAI, CUS, O	11.1	0.9	9.6	TA, SM
*Rumex crispus L.*	Polygonaceae	Romaza, lengua de vaca	Ex	Water bodies	GI, F	22.2	0.4	9.6	NO
*Tetraglochin alatum* (Gillies ex Hook. & Arn.) Kuntze	Rosaceae	Yerba de la perdiz	Na	Dry plates	GI, OG, GU	16.7	0.7	12.3	TA
*Artemisia abrotanum* L.	Asteraceae	Buen viejo, éter	Ex	Anthropized areas	GI	27.8	0.3	8.5	TA, SM
*Azorella monantha* Clos	Apiaceae	Leña de piedra	Na	Dry plates	GI	27.8	0.3	8.5	TA, SM
*Larrea* sp.	Zygophyllaceae	Jarilla	Na	Dry plates	AAI, OG	11.1	0.4	4.8	TA, SM
*Mentha spicata* L.	Lamiaceae	Menta, yerba buena	Ex	Water bodies	GI, RE	27.8	0.4	12.0	TA, SM
*Mentha aquatica* L.	Lamiaceae	Menta canela, menta, menta negra	Ex	Cultivated areas	GI	22.2	0.2	4.8	TA, SM
*Polygonum aviculare* L.	Polygonaceae	Sanguinaria	Ex	Anthropized areas	CV, GU	11.1	0.4	4.8	TA
*Schinus patagonicus* (Phil.) I.M. Johnst. ex Cabrera	Anacardiaceae	Laura	Na	Water bodies	RE, CUS	11.1	0.4	4.8	TA, SM
*Allium sativum* L.	Alliaceae	Ajo	Ex	Purchase/barter	GI, AAI	5.6	0.5	2.9	TA, SM
*Plantago* sp.	Plantaginaceae	Siete venas	Ex	Anthropized areas	DC, O	5.6	0.3	1.9	NO
*Schinus* roigii Ruiz Leal & Cabrera	Anacardiaceae	Molle	Na	Water bodies	RE, DC, CUS	11.1	0.7	8.2	TA
8^c^	?	Mata dolor	?	Dry plates	RE, AAI	16.7	0.4	7.2	TA
11^c^	?	?	?	Stony slopes	GI, RE, AAI	5.6	0.6	3.6	TA
*Malva sylvetris* L.	Malvaceae	Malva	Ex	Anthropized areas	GI, O, GU	11.1	0.6	7.2	NO
*Oenothera odorata* Jacq.	Onagraceae	Yerba de san Juan	Na	Dry plates	?	16.7	0.2	3.6	TA
*Origanum vulgare* L.	Lamiaceae	Orégano	Ex	Purchase	RE, OG, O	11.1	0.6	7.2	TA, SM
*Senecio* sp. 1	Asteraceae	Charkaw^b^	Na	Dry plates	RE, AAI	16.7	0.4	7.2	TA, SM
*Viola maculata* Cav.	Violaceae	Oreja de ratón	Na	Dry plates	GI	11.1	0.5	5.4	NO
*Lycium chilense* Miers ex Bertero	Solanaceae	Monte negro	Na	Dry plates	OG	11.1	0.4	4.4	TA
*Mutisia retrorsa* Cav.	Asteraceae	Redadera	Na	Water bodies	RE	5.6	0.3	1.7	TA, SM
9^c^	?	Monte verde	?	Stony slopes	GI, CUS	5.6	0.4	2.4	NO
10^c^	?	?	?	Dry plates	GI	11.1	0.2	2.4	TA, SM
13^c^	?	?	?	Purchase/barter	GI, CUS	5.6	0.4	2.4	NO
4^c^	Poaceae	Pasto de perro	?	Anthropized areas	GI, F	5.6	0.4	2.4	NO
6^c^	?	Flor de piedra	?	Stony slopes	GI, CUS	5.6	0.4	2.4	NO
*Discaria* sp.	Rhamnaceae	Chakay^b^	Na	Water bodies	GI, RE	5.6	0.4	2.4	NO
*Gamocarpha selliana* Reiche	Calyceraceae	Pata de williñ^b^	Na	Water bodies	GI, CUS	5.6	0.4	2.4	NO
*Mentha* suaveolens Ehrh.	Lamiaceae	Menta chilena	Ex	Cultivated areas	GI, DC	5.6	0.4	2.4	TA, SM
*Nardophyllum bryoides* (Lam.) Cabrera	Asteraceae	Siete camisas	Na	Dry plates	GI	11.1	0.2	2.4	TA, SM
*Nassauvia glomerulosa* (Lag. ex Lindl.) D. Don	Asteraceae	Uña de gato, cola de piche	Na	Dry plates	O	11.1	0.2	2.4	NO
*Oryza sativa* L.	Poaceae	Arroz	Ex	Purchase	GI	11.1	0.2	2.4	TA
*Peumus boldus* Molina	Monimiaceae	Boldo	Ex	Purchase/barter	GI	11.1	0.2	2.4	TA, SM
*Quillaja saponaria* Molina	Quillajaceae	Palo jabón, jabón de palo	Ex	Purchase	CV	11.1	0.2	2.4	TA, SM
*Rosa rubiginosa* L.	Rosaceae	Rosa mosqueta	Ex	Anthropized areas	RE, O	5.6	0.4	2.4	TA
*Sanicula graveolens* Poepp. ex DC.	Apiaceae	Cilantro de campo, cilantro silvestre	Na	Water bodies	GI	11.1	0.2	2.4	TA, SM
*Senecio* sp. 2	Asteraceae?	Paco	Na?	Stony slopes	DC, F	5.6	0.4	2.4	SM
*Tilia* sp.	Tiliaceae	Tilo	Ex	Purchase	GI, CUS	5.6	0.4	2.4	TA
*Valeriana clarionifolia* Phil.	Valerianaceae	Ñamkulawen^b^	Na	Stony slopes	CV, AAI	5.6	0.4	2.4	TA, SM
*Verbascum thapsus* L.	Scrophulariaceae	Tabaco, tabaco de turco	Ex	Anthropized areas	?	11.1	0.2	2.4	SM
*Populus nigra* L.	Salicaceae	Álamo	Ex	Cultivated areas	GI	5.6	0.4	2.2	NO
1^c^	?	Füre lawen^b^	?	Dry plates	RE	5.6	0.2	1.2	TA, SM
12^c^	?	?	?	Dry plates	DC	5.6	0.2	1.2	NO
2^c^	Asteraceae	Cardo	Ex	Anthropized areas	O	5.6	0.2	1.2	TA
3^c^	?	?	?	Dry plates	GI	5.6	0.2	1.2	NO
5^c^	?	Poñí cacho^b^	?	Dry plates	DC	5.6	0.2	1.2	NO
7^c^	Juncaceae	Unquillo	?	Water bodies	DC	5.6	0.2	1.2	NO
*Aloe* sp.	Xanthorrhoeaceae	Aloe	Ex	Cultivated areas	GU	5.6	0.2	1.2	NO
*Aristotelia chilensis* (Molina) Stuntz	Elaeocarpaceae	Make^b^	Na	Barter	GI	5.6	0.2	1.2	NO
*Beta vulgaris* L.	Chenopodiaceae	Acelga	Ex	Cultivated areas	O	5.6	0.2	1.2	TA
*Calceolaria uniflora* Lam.	Calceolariaceae	?	Na	Dry plates	RE	5.6	0.2	1.2	NO
*Camellia sinensis* (L.) Kuntze	Theaceae	Té común	Ex	Purchase	O	5.6	0.2	1.2	TA, SM
*Conium maculatum* L.	Apiaceae	Cicuta	Ex	Anthropized areas	O	5.6	0.2	1.2	SM
*Euphorbia collina* Phil.	Euphorbiaceae	Pichoga	Na	Dry plates	GI	5.6	0.2	1.2	NO
*Ilex paraguariensis* A. St. -Hil.	Aquifoliaceae	Yerba mate	Ex	Purchase/barter	OG	5.6	0.2	1.2	TA
*Iris* sp.	Iridaceae	Lirio blanco	Ex	Cultivated areas	CUS	5.6	0.2	1.2	NO
*Laurus nobilis* L.	Lauraceae	Laurel	Ex	Purchase	AAI, CUS	5.6	0.2	1.2	TA, SM
*Lomatia hirsuta* (Lam.) Diels	Proteaceae	Radal	Na	Stony slopes	AAI	5.6	0.2	1.2	NO
*Maytenus boaria* Molina	Celastraceae	Maitén	Na	Barter	CUS	5.6	0.2	1.2	NO
*Mentha* sp.	Lamiaceae	Pepermina	Ex	Cultivated areas	GI	5.6	0.2	1.2	TA
*Mutisia decurrens* Cav.	Asteraceae	Redadera	Na	Water bodies	?	5.6	0.2	1.2	TA
*Nasturtium officinale* W. T. Aiton	Brassicaceae	Berro	Ex	Water bodies	GI	5.6	0.2	1.2	TA
*Potentilla chiloensis* (L.) Mabb.	Rosaceae	Frutilla	Na	Water bodies	O	5.6	0.2	1.2	TA
*Rhodophiala* mendocina (Phil.) Ravenna?	Amaryllidaceae	Juanita	Na	Dry plates	OG	5.6	0.2	1.2	TA
*Salvia officinalis* L.	Lamiaceae	Salvia	Ex	Cultivated areas	OG	5.6	0.2	1.2	TA, SM
*Saponaria officinalis* L.	Caryophyllaceae	?	Ex	Water bodies	DC	5.6	0.2	1.2	TA, SM
*Schinopsis lorentzii* (Griseb.) Engl.	Anacardiaceae	Quebracho colorado	Ex	Purchase/barter	AAI	5.6	0.2	1.2	NO
*Solanum* sp.	Solanaceae	Tomatito	Na	Anthropized areas	DC	5.6	0.2	1.2	TA, SM
*Tanacetum balsamita* L.	Asteraceae	Menta	Ex	Cultivated areas	?	5.6	0.2	1.2	TA, SM
*Taraxacum officinale* G. Weber ex F.H. Wigg	Asteraceae	Chicoria	Ex	Water bodies	O	5.6	0.2	1.2	TA
*Tristagma patagonicum* (Baker) Traub	Alliaceae	Cebolla de campo	Na	Dry plates	?	5.6	0.2	1.2	TA
*Triticum aestivum* L.	Poaceae	Trigo	Ex	Purchase/barter	GI	5.6	0.2	1.2	TA
*Tropaeolum incisum* (Speg.) Sparre	Tropaeolaceae	?	Na	Stony slopes	RE	5.6	0.2	1.2	SM
*Urtica magellanica* Poir.	Urticaceae	Ortiga	Na	Stony slopes	?	5.6	0.2	1.2	NO

^a^Lichen.

^b^Mapuzungun name.

^c^Undetermined species.

References: biogeographic origin: Ex = exotic, Na = native; medicinal uses: GI = gastro-intestinal, RE = respiratory, AAI = analgesic-anti-inflammatory, DC = dermatological-cosmetic, CUS = cultural syndromes , F = feber, CV = cardio-vascular, OG = obstetric-gynaecological, GU = genito-urinary, and O = other uses; RI = relative importance; sensorial property: TA = taste, SM = smell, NO = no taste-no smell.

Gathering in particular is carried out in areas where the successional management of fodder plants and shrubs is considered a priority. This practice increases the frequency of visits to the different herbal landscape units, enabling verification of the presence of resources and changes in their availability, as well as the perception of their colors, flavors and aromas. Plants resources are collected in quantities suitable for family use alone, principally in zones close to water bodies, which include rivers and waterholes known as “menucos” or “aguadas”, and flood meadows or “mallines” of glacial origin (23%). On some family lands, other management practices have been observed, such as the diversion and/or isolation of these water bodies for the protection of medicinal, edible or fodder species of high cultural value. The importance of these peri-aquatic environments as sources of medicinal resources is likely to be a result of their high levels of biodiversity [31], a characteristic valued by local populations [32].

To a lesser extent, gathering is also carried out on the meseta and plains, known as “pampas” and “lomas secas” (21.5%) where the stock animals are grazed for most of the year; and on the stony slopes “pedreros de la cordillera” and “nevados” (a high Andean phytogeographic environment) (18%). This last mentioned region is visited by inhabitants who still maintain the practice of summer sheep and goat pasturing (around 50% of family heads). Various studies have reported that this summer pasturing constitutes a form of management that allows pastures to rest, and implies successional herb management, which favors more efficient use of weeds spread by the animals [18].

Another environment worthy of note is the area surrounding the dwellings, described in the interviews as “just around here”, and other anthropized sites such as the verges of main roads and country roads, paddocks and corrals (16%). Taking advantage of these readily available and easily accessible ruderal species [19,33] implies a kind of management, due to the link between their dispersion and human activities. Particularly, in this environment, an interesting case is the use of *Schinopsis lorentzii* (Griseb.) Engl. (red quebracho). This species is native to the Chaco region of NE Argentina, and is sold in health food shops and pharmacies in Argentina due to its well-known anti-diarrhoea, emollient, antiseptic and tissue healing powers [34]. Nevertheless, in Nahuelpan this species is obtained mainly by gathering, in this case by the scraping of fence posts or railway track sleepers made of this wood. Thus, the inhabitants take advantage of the cultural landscape in multiple, original ways, according to local availability.

With regard to the eco-sensoriality of these herbal landscape units, it was found that apart from in the “pampas” and “lomas secas”, most medicinal plants belonging to remaining environments are basically defined, characterized and valued according to their taste (binomial test, *p* <0.05). In contrast, with respect to their aromaticity, only in the “pedreros” and “nevados de la cordillera” did aroma figure as a differential trait in most of the plants (binomial test, *p* <0.05).

However, these units are not isolated and present a certain amount of spatial continuity, not only with respect to ecological gradient but also to the many interconnections generated from the network of roads and tracks established and used by the inhabitants themselves and their domestic animals. Because of this, their limits are not clear-cut, but rather diffuse and porous [7]. According to ethnobotanical approach the physical spaces of cultural landscape are scenarios that reflect an intricate network of people, places and resources over time, so that their boundaries are necessarily dynamic in response to different needs, ideas and cultural practices [2]. In particular, these paths often guide the movements of the shepherds and their flocks, having been tread and re-tread in response to the differential availability of pastures in different years, and the establishment or abandonment of dwellings. These interconnectors themselves favor the exchange of medicinal plants among neighbors, as well as activities associated with other products such as sheep’s fleece, tools, food, etc.

### Barter and purchase and their relation to the herbal landscape

The social and economic activity of bartering fulfills an important role in the recreation of the Nahuelpan plant landscape. This practice favors the periodic interaction of families within the community and with others from neighboring communities, or rather, with workers who return from the sheep shearing operations on other Patagonian ranches.

By means of barter, other species from distant environments, which are mostly aromatic and sapid, are added to the repertory of plants used (binomial test, *p* <0.05; Table 1). Among these, worthy of special mention are *Larrea* sp. (“jarilla”) native to the Patagonian Monte, *Solanum crispum* Ruiz et Pav.(“natre”) native to sub Antarctic forests, and *Eucalyptus* sp. (“eucalito”), an exotic species native to Australia, planted widely along the Atlantic coast that is commonly gather by rural workers in the ranches.

Bartering is complemented with purchasing, an additional strategy which allows the acquisition of fragmented, packaged or loose medicinal resources in grocer’s, health food shops and pharmacies in the town of Esquel, or from travelling salespeople. Some authors suppose that this practice could indicate disuse of gathering, or even processes of erosion of traditional knowledge [35]. However, in this case study, purchase as a supply strategy indicates processes leading to the expansion and/or diversification of the therapeutic cultural landscape [6]. In this sense, the incorporation of resources exogenous to Nahuelpan could be associated with an adaptive strategy to deal with a serious environmental circumstance, such as the growing regional process of desertification which limits the availability of some of the repertory of plants the people need (e.g. *Matricaria recutita* L. and *Gunnera tinctorea* (Molina) Mirb*.*).

All the plants purchased are defined and characterized by the presence of flavor and aroma (binomial test, *p* <0.05).

### Homegardens, pot plants and the herbal landscape

The herbal landscape also includes the homegardens constructed by inhabitants, modifying the appearance of the location, and constituting food and medicinal plant supply sites. These plant sources are frequently situated in peridomestic areas. Using this strategy, fourteen species of exotic medicinal species (e.g. *Melissa officinalis* L., *Mentha* spp., *Beta vulgaris* L., *Salvia officinalis* L. and *Tanacetum balsamita* L.) are harvested, which is a low number for the inhabitants, and a consequence of a lack of irrigation water, although the progressive loss of ancestral domestic horticultural practices could also be a cause [20]. The majority of these plants are for alimentary use, and originated from packet seed provided by INTA.

In these cultivation sites preexisting native species in the homegardens are tolerated and/or protected, coexisting with the cultivated ones, as is the case of the shrubs *Corynabutilon bicolor* (Phil. ex K. Schum.) Kearney and *Fabiana imbricata* Ruiz et Pav., which are also valued as hedges, for protection and shelter from the wind and sun, and refuge for farm animals. Following [5,36], tolerance is a common management practice here so which these plants are left deliberately standing when vegetation is disturbed; in addition, people make actions of protection favoring permanence of these plants through special care (pruning, elimination of weeds, etc). However, no evidence was found in homegardens of other management practices with this native medicinal plants, such as transplanting or planting seed or propagules from wild areas.

Other species such as *Populus* spp.*, Sambucus nigra* L., *Tanacetum vulgare* L. and *Iris* spp., are cultivated principally for their ornamental value and as shelter from the wind, and occasionally used for medicinal purposes (Table 1). The fact that these are multifunctional species seems to be a very important selection criterion in rural communities. It has been found that in hedge species, their additional alimentary and/or medicinal value constitutes a notable trait in rural populations of Patagonia [37,38]. However, there are also other medicinal species used which are important due to their uniqueness and effectiveness, such as *Salvia officinalis* L. for obstetric-gynaecological diseases and *Mentha aquatica* L. for gastro-intestinal pains.

Three exotic species are cultivated in pots: *Sedum telephium* L., *Ruta chalepensis* L. (both outdoors) and *Aloe* sp. (indoors), which are, in general, plants valued for their therapeutic and symbolic prestige, whose use extends to other Mapuche communities in the region [25]. These are plants which also adorn dwellings, but their principal symbolic function is to act as protectors or defensive elements and purifiers of the domestic environment. The cultivated species are mostly cited with reference to their sapid qualities (binomial test, *p* <0.05), while aromaticity is not a feature that characterizes them significantly (binomial test, *p* >0.05).

### The cultural importance of plant landscape units

The highest average values for cultural importance were obtained for the species found in high environments, on the stony slopes or “pedreros de la cordillera” (average consensus value * RI = 29) followed by the water bodies: “arroyos”, “menucos” and “mallines” (25), while the lowest values were obtained for the units close to dwellings and other anthropized zones (20), for the dry plates: “pampas” and “lomas secas” (11), for “purchase” and “barter” (10) and for the cultivated areas (9). However, the comparative analysis of these values indicates no significant differences (Kruskal-Wallis test, p = 0.65). One relevant characteristic of the principal species, in terms of the indices considered, whether gathered, purchased, bartered or cultivated, is that they are aromatic and/or sapid (Table 1).

The higher cultural importance of species coming from high Andean environments coincides with reports on other Andean populations in the southern cone [33,39]. This speaks of the great therapeutic versatility of these environments, which could explain their high cultural value. Molares and Ladio [25] have highlighted the sacred character and the higher medicinal potential of the highest altitude levels for other Mapuche communities of Argentina.

### Medicinal species: characteristics and organoleptic classification

Nahuelpan inhabitants mentioned 121 ethnospecies belonging to 55 botanical families, of which 108 were identified taxonomically (Table 1). The most frequently cited species were: *Baccharis sagittalis* (Less.) DC (“carqueja”) (83.3%), *Gunnera tinctorea* (Molina) Mirb. (“nalca”) (83%), *Artemisia absinthium* L. (“ajenco”) (72%), *Fabiana imbricata* Ruiz. & Pav. (“palo piche”) (72.2%), *Adesmia boronioides* Hook. f. (“paramela”) (66.7%), *Marrubium vulgare* L. (“malva rubia”) (55%) and *Valeriana carnosa* Sm. (“ñamkulawen”) (66.7%); all these species are strongly aromatic and/or sapid.

Considering only the data on the taxonomically identified species, it was found that native species richness (59) was higher than that of exotic species (49) (binomial test, *p* <0.05). In recent years the use of exotic species has begun to be interpreted as possibly reflecting a certain amount of flexibility on the part of individuals in order to take advantage of the changes taking place in their cultural landscape, as they incorporate new resources that contribute to their survival and wellbeing. This would indicate exploratory processes with unknown or recently discovered plants, demonstrating a certain capacity for adaptation on the part of local inhabitants [40].

Of the inhabitants interviewed, 80% mentioned that all the medicinal plants had an aroma, and that if an aromatic plant was not recognized as medicinal it was very likely that it hadn’t been tested to identify its properties.

Of a total of 456 positive recordings of the presence of organoleptic characteristics of the mentioned species, it was found that 60% corresponded to flavor and 40% to aroma. There are no organoleptic data for 34% of the species registered. This could be due to the taboo associated with communicating certain symbolic aspects of traditional botanical knowledge to people from outside the community, in particular because certain strong aromas serve as vehicles or intermediaries between the natural and supernatural worlds [29].

Of the plants cited, 78 species are described by their flavor and 52 by their aroma (Table 1). In general terms informants classified the flavor as “acid”/“sour” (e.g., the whole plant of *Oxalis* spp.), “bitter” (e.g., the stalks of *Baccharis sagittalis* (Less.) DC), “spicy” (e.g., the roots of *Lycium chilense* Miers ex Bertero), “sweet” (e.g., the branches of *Ribes cucullatum* Hook. & Arn*.*), and “dry”/“like alum”/“like tea” (referring to *Camellia sinensis*, e.g., the roots of *Acaena splendens* Hook. & Arn*.*) (Table 1). The last mentioned character refers to the perception of astringency, which, according to Amat and Vincent [41] is a property perceived more by the sense of touch than of taste.

Aroma is perceived as “scented” (e.g. branches of *Acantholippia seriphioides* (A. Gray) Moldenke), “minty” (referring to *Mentha* spp., e.g. branches of *Clinopodium darwinii* (Benth.) Kuntze), “like boldo” (referring to *Peumus boldus* Molina, e.g. branches of *Dysphania ambrosioides* (L.) Mosyakin & Clemants), “like tobacco” (referring to *Nicotiana tabacum L.*, e.g. leaves of *Verbascum thapsus L.*), “like the smell of dirty feet” (e.g. roots and rhizomes of *Valeriana carnosa* Sm.), “heavy, dense” (e.g. branches of *Ruta chalepensis* L.), “like caramel”, because of the aroma and resinous texture (e.g.: stalks of *Adesmia boronioides* Hook. f.).

In addition, in many cases aroma and flavor were interpreted together, and subclassified into two contrasting categories: “füre” (strong/unpleasant/disgusting, in Mapuzungun) (e.g. branches of *Artemisia absinthium* L.) and “mild/nice” (e.g. roots of *Armeria maritima* (Mill.) Willd.). In the interviews, informants were not able to distinguish between unpleasant and strong, or between mild and nice. This indistiction between the quality and the intensity of aromas and flavors may indicate the absence of linguistic labels for these perceptions [42], or possibly a fundamentally practical perceptual-functional connection.

### Therapeutic uses and sensorial characteristics

In total, 526 reports of medicinal uses were registered. Of these, 25% were related to gastro-intestinal use, 19% to respiratory, 10% to analgesic-anti inflammatory, 9% to dermatological-cosmetic and 8% to cultural syndromes (“mal de ojo”, “pasmo”, “frío”, “mal aire”), 6% to febrifuges, 6% to cardio-vascular, 6% to obstetric-gynaecological, 5% to genito-urinary and 6% to other uses (endocrine, ophtalmological, etc.) (*p* <0.05).

It is noteworthy that certain species with mild aromas and/or flavors, recognized as “kochü lawen”, are used for strongly culturally based syndromes. For example, *Erodium cicutarium* (L.) L’Hér. ex Aiton, a plant which has become established in the wild and is widespread in anthropized areas throughout the region, is used to treat the “pasmo”, a condition that includes symptoms such as stomach pains, cramps and abnormal pigmentation of the skin, which is caused by an imbalance between the states of hot and cold; the cultivated, aromatic *Melissa officinalis* L. is used for “afligimiento del corazón (heart troubles)” which involves feeling sadness and a general decline in spirits; the purchased seeds of *Linum usitatissimum* L.are used after childbirth (for “domo kütran” or “mollfüñ kütran”) which is “cuando la sangre queda coajada adentro (when coagulated blood remains inside)”, a problem which presents symptoms of headaches, backaches and bleeding after the birth, and can be fatal. There are also certain problems related to the supernatural world, such as the “susto” (fright), the result of a meeting with spirits such as the “anchimallen” (who takes the form of a child), with the “cherrufes” (lights in the countryside), with the “meulen” (dangerous whirlwinds), or with the “cuero” or “wayllepen” (an animal with extended limbs which appears in isolated places close to water bodies), among others, which often require highly aromatic species in their treatment. These belong to the group of the “alwe lawen” (medicines for the souls of the dead), and include species such as *Rosmarinus officinalis* L., *Ruta* spp., *Laurus nobilis* L. and *Artemisia absinthium* L., which are considered “füre lawen” (strong, bitter, potent medicines). These species are generally used in the form of aromatic smoke, thrown on the embers of the fire; hung on the body as amulets beneath the clothing, on the walls of dwellings, or on the door thresholds as protectors or ‘defensive’ elements. They also tend to be grown in pots or in homegardens so as to favor the wellbeing of the family and their animals.

In order to deal with these and many other problematic situations, the informants have restructured the significance of resources which have appeared relatively recently in the region. These processes show, in part, the adaptive capacity of these indigenous communities, which comes into action when socio-cultural and ecological changes must be faced [18].

Similar to that detected in the studies carried out in communities of the sub Antarctic forest [8,25], it was found that the plants with highest use consensus, in this case used for digestive, respiratory, cardio-vascular, analgesic-anti-inflammatory, obstetric-gynaecological and genito-unrinary complaints, have the highest frequencies of cites reporting flavor (Spearman test, *p* <0.05), and those with the highest frequencies relating to digestive, analgesic-anti-inflammatory and cultural syndromes present the highest frequencies of cites reporting aroma (Spearman test, *p* <0.05; Table 1).

## Conclusions

This work on the cultural landscape of a community living in the steppe, studied from an eco-sensorial perspective, sheds new light on the ways societies perceive and manage their natural surroundings. The pattern found in this study shows that a bidirectional process is occurring; local people activities on their arid land and the subsequent environment respond are very closely connected, possibly adding, a specific organoleptic trait in each landscape unit.

Our focus on the medicinal plants available in the community enabled us to discover that the processes of cultural perception, use, selection and management of these surroundings are influenced by the organoleptic attributes of their components. These components undergo changes, that is, native and exotic wild species, species which are bartered or purchased and those cultivated in the homegardens may all enter or leave the system, depending on environmental and social circumstances that regulate the everyday lives of the inhabitants. Barter and purchase, as socially extended practices of high cultural value, are strategies and relevant sources for the supply of medicinal resources, which extends the limits of the herbal landscape, in such a way that other layers are added to the local medical system, which, although they do not occupy any defined area of land, expand the herbal landscape as a whole [6]. Different social-political and environmental phenomena over the last century have had an impact on the Patagonian biocultural landscape [36,43]. In this complex landscape, changeable in its components and processes, knowledge of the resources is learned in the everyday business of survival, both individually and collectively, indoors and outdoors, and is permeable to the flow of exogenous information [18,44]. Access to the mass media, formal education, the involvement of external social organizations, and the inclusion of more young people in the labor markets of towns in the region have favored the incorporation of knowledge and resources proceeding from the global market.

The pharmacopeia as a cultural construction of the local medical system has not been excluded from this context, but has adjusted to it through innovatory processes [45], thus providing more effective answers to health problems in its prophylactic and therapeutic role and in the diversity of psycho-physical and emotional circumstances that arise.

In this process of change, organoleptic perceptions play a significant role in the criteria of identification, selection and use of medicinal plants [2,9,25], which are also fluctuating in the time and space.

According to various authors, the association between chemo-sensorial concepts and precepts and particular therapeutic functions is an underlying cognitional strategy that operates, together with other mental processes, as a heuristic tool [46-48]. It has been proposed that the heuristic part is the simple rule that guides the making of decisions and resolves practical problems. In general, a heuristic process can be considered as a short cut in complex mental processes, and is therefore a measure which economizes on mental resources while frequently arising from analogical reasoning [49]. An example of a mental short cut is the use of a stereotype or prototype, that is, an element that best expresses the characteristics of a group of elements belonging to a culturally significant domain, such as a category of medicinal use [46]. When a species is judged according to a stereotypic description of a group it is thought to belong to, the use of the stereotype can be useful. In this sense, organoleptic perceptions can be valuable features in the characterization of plant prototypes [48]. The importance of species prototypes lies in their role as cultural and personal reference points, which help individuals remember and transmit knowledge of illnesses and their treatment to other members of the population [48]. Thus, prototypes may function as brief guides in the selection of species from the flora with which each population interacts on a daily basis [11]. In this study we can show that, organoleptic perception help people in the determination of the functions of new available resources, and also in the assimilation of ideas and symbols different to their native ones. All these perceptive processes form part of the construction of cultural landscapes, and our study is crucial for a more in depth understanding of their dynamics in time and mechanisms of adaptation to deal with the socio-environmental changes faced by indigenous communities today.

## Competing interest

The authors declared that they have no competing interests.

## Authors’ contribution

SM and AL have conceptualized the study and wrote the entire manuscript. Both authors approved the final version of the manuscript.
